# Hospital Stay as a Proxy Indicator for Severe Injury in Earthquakes: A Retrospective Analysis

**DOI:** 10.1371/journal.pone.0061371

**Published:** 2013-04-09

**Authors:** Lu-Ping Zhao, Martin Gerdin, Lina Westman, Jose Manuel Rodriguez-Llanes, Qi Wu, Barbara van den Oever, Liang Pan, Manuel Albela, Gao Chen, De-Sheng Zhang, Debarati Guha-Sapir, Johan von Schreeb

**Affiliations:** 1 People's Hospital of Deyang City, Deyang, Sichuan province, China; 2 Health System and Policy, Department of Public Health Sciences, Karolinska Institutet, Stockholm, Sweden; 3 Höglandssjukhuset, Eksjö, Sweden; 4 Centre for Research on the Epidemiology of Disasters, Institute of Health and Society, Université catholique de Louvain, Brussels, Belgium; University of Toronto, Canada

## Abstract

**Introduction:**

Earthquakes are the most violent type of natural disasters and injuries are the dominant medical problem in the early phases after earthquakes. However, likely because of poor data availability, high-quality research on injuries after earthquakes is lacking. Length of hospital stay (LOS) has been validated as a proxy indicator for injury severity in high-income settings and could potentially be used in retrospective research of injuries after earthquakes. In this study, we assessed LOS as an adequate proxy indicator for severe injury in trauma survivors of an earthquake.

**Methods:**

A retrospective analysis was conducted using a database of 1,878 injured patients from the 2008 Wenchuan earthquake. Our primary outcome was severe injury, defined as a composite measure of serious injury or resource use. Secondary outcomes were serious injury and resource use, analysed separately. Non-parametric receiver operating characteristics (ROC) and area under the curve (AUC) analysis was used to test the discriminatory accuracy of LOS when used to identify severe injury. An 0.7<AUC<0.8 was defined as adequate.

**Results:**

Our study shows that LOS discriminatory accuracy is poor for the primary outcome. However, LOS discriminatory accuracy is adequate for resource use, excluding critical orthopaedic interventions and debridement.

**Conclusions:**

Length of hospital stay was not validated as a proxy indicator for severe injury in earthquake survivors. However, LOS was found to be a proxy for major nonorthopaedic surgery and blood transfusion. These findings can be useful for retrospective research on earthquake-injured patients when detailed hospital records are not available.

## Introduction

The ten most deadly earthquakes during the last century killed more than 1.4 million people. Four of those earthquakes occurred in China and one each in Indonesia, Japan, the Soviet Union, Italy, Pakistan, and Haiti [1]. Earthquakes affect human health directly by causing traumatic injuries and indirectly by disrupting the health care systems. The dominating medical problem in the early phase after earthquakes is injuries [2]. The early phase after earthquakes is characterized by chaos and data-collection is not a top priority. Thus, research on injuries due to earthquakes is lacking and anecdotal reports from individual health care providers rather than systematic research dominate the literature [3].

Retrospective injury research depends largely on quantification of injury severity, but adopting a common scoring system for injury severity in a post-earthquake setting may not be feasible. Nevertheless, information about injury severity is important and finding a proxy indicator for injury severity is therefore desirable. This proxy indicator should have a clear definition; its collection should minimally distract from patient care and should not require medical training. In addition, the proxy indicator should be universally accepted as an important part of routine data collection regardless of setting.

Length of hospital stay (LOS) fulfils these criteria and has been validated as a precise proxy indicator for injury severity in non-disaster and high-income settings [4]. However, it has not been validated as a proxy indicator for injury severity in an earthquake setting. In this study, we assessed LOS as an adequate proxy indicator for severe injury in earthquake survivors.

## Materials and Methods

A retrospective study was conducted on data from 1,878 injured patients admitted to People's Hospital of Deyang City (PHDC), China, after the 2008 Wenchuan earthquake. The data set is part of collaboration between the PHDC, the Centre for Research on the Epidemiology of Disasters (CRED), Belgium, and Karolinska Institutet (KI), Sweden. This study was approved by the Ethical Committee of People's Hospital of Deyang City. The need for informed consent was waived as the database was retrospectively collated from already existing data.

### Setting

On May 12, 2008, an earthquake measuring 7.9 on the Richter scale hit Wenchuan county, Sichuan province, China. The Sichuan province is one of the most industrialized and densely populated areas in China, with a wide range of agricultural and industrial activities. The earthquake's epicentre was located in Wenchuan County, about 80 km from Chendgu, the capital of the Sichuan province. About 348 million people across eight states and 857 counties were affected by the quake. Reports released by the Ministry of Civil Affairs of China eventually reported 69,227 people killed; 17,923 missing; and 374,643 injured, along with the displacement of about 15 million people [5].

PHDC is the largest level 3 state-owned hospital in Deyang district and was the closest hospital to the epicentre, 99 kilometres. With a capacity of 1,200 beds the hospital has a service area covering a population of four million. The number of working staff was 1,462, with 435 doctors, 743 nurses and 130 paramedical personnel. In terms of hospital structure, the PHDC has 30 medical departments, 10 technical sections, 20 inpatient sectors, 1 intensive care unit (ICU) and 4 clinical departments of provincial importance, including orthopaedics, general surgery, neurosurgery and neurology. During 2007, the year before the earthquake, the average LOS for patients in neurosurgery and orthopaedics was 17 days.

Although the earthquake damaged the hospital structurally, no crucial diagnostic equipment was impaired and hence PHDC was able to provide health care free of charge immediately after the earthquake. The hospital was not affected by power outage. Some roads to the hospital were damaged; and roads nearby the hospital suffered from traffic jams, which indirectly affected the hospital's medical activity. All in-hospital patients were transferred to an open yard in preparation for after-shocks. PHDC being the largest public hospital close to the epicentre with a level of functioning that allowed comprehensive record keeping together made up the rationale for conducting research on earthquake-related injuries here.

### Development of the database

Immediately after the earthquake, PHDC organized a dedicated medical team working with patient registration. People with injuries were brought to the hospital mainly by family members, friends, or co-workers. Whether a person's injuries were earthquake-related or not was established by asking the patient, if he or she was conscious, or his or her accompanies. Researchers from PHDC and CRED later developed the database to study the medical effects of the earthquake, and hence only patients with earthquake-related injuries were included. Initially, patient files were translated from Mandarin to English. All variables available from the patient files were listed in a database as jointly decided by CRED researchers and PHDC medical experts. A total of 52 variables were selected for inclusion, and then defined in a codebook. The selected variables included information on patient demographics, admission and discharge, up to five injury diagnoses (each with corresponding ICD-codes), surgical interventions, and other procedures performed (with corresponding ICD-9-CM-3-codes).

Trained operators then extracted data between March 12 and May 5, 2010, from files of patients admitted to PHDC between May 12 and May 31, 2008. In total, 1,950 patients with earthquake-related injuries were admitted to PHDC during the first 19 days following the earthquake. Of these, 72 were excluded from our study due to missing data on key variables. The final dataset used for this study included 1,878 patients.

### Eligibility criteria

Patients were excluded from the final analysis if they did not survive until discharge; were transferred; had any of the diagnoses recorded in the database that were non-traumatic, or a procedure code; or if data on age, sex, or LOS were missing. All patients were included in sensitivity analysis to test the effect of including versus excluding patients with certain characteristics.

### Variables

The predictor variable was LOS, defined as number of days from admission to discharge. The primary outcome variable was severe injury, defined as a composite measure of serious injury or resource use (Table 1). We used this primary outcome for several reasons. Firstly, previous research has shown that by using only injury severity scores to define severe injury one risks missing patients that for some reason score low but still are severely injured. Including resource use in the definition of severe injury is supposed to correct this shortcoming [6]. For example, a patient that scores above the cut-off for serious injury but still receives an acute craniotomy must be assumed to be severely injured. Secondly, we wanted to be able to compare our results with previous research in which this composite measure has been used [4].

**Table 1:** Outcomes and definitions.

Outcome	Type	Definition
Severe injury	Primary	Serious injury (ICISS<0.9) and/or major surgery (brain or spine, thoracic, abdominal, neck, vascular, debridement under general anaesthesia, or critical orthopaedic surgery) or blood transfusion
Severe injury excluding critical orthopaedic surgery from resource use definition	Secondary	Serious injury (ICISS<0.9) and/or major surgery (brain or spine, thoracic, abdominal, neck, vascular, or debridement under general anaesthesia) or blood transfusion
Severe injury excluding debridement under general anaesthesia from resource use definition	Secondary	Serious injury (ICISS<0.9) and/or major surgery (brain or spine, thoracic, abdominal, neck, vascular, or critical orthopaedic surgery) or blood transfusion
Severe injury excluding critical orthopaedic surgery and debridement under general anaesthesia from resource use definition	Secondary	Serious injury (ICISS<0.9) and/or major surgery (brain or spine, thoracic, abdominal, neck, or vascular) or blood transfusion
Resource use	Secondary	Major surgery (brain or spine, thoracic, abdominal, neck, vascular, debridement under general anaesthesia, or critical orthopaedic surgery) or blood transfusion
Resource use excluding critical orthopaedic surgery	Secondary	Major surgery (brain or spine, thoracic, abdominal, neck, vascular, or debridement under general anaesthesia) or blood transfusion
Resource use excluding debridement under general anaesthesia	Secondary	Major surgery (brain or spine, thoracic, abdominal, neck, vascular, or critical orthopaedic surgery) or blood transfusion
Resource use excluding critical orthopaedic surgery and debridement under general anaesthesia	Secondary	Major surgery (brain or spine, thoracic, abdominal, neck, or vascular) or blood transfusion
Serious injury	Secondary	ICISS<0.9

ICISS  =  ICD-derived Injury Severity Score.

Serious injury was defined as an ICD-derived injury severity score (ICISS) below 0.90. This cut-off has also been used to define seriously injured patients in previous studies [4], [7]. We used ICISS because it has been showed to perform well compared to other established injury severity measures such as the Injury Severity Score (ISS) and the Trauma and Injury Severity Score (TRISS) [8]. In addition, ICISS is easily computed if all injuries are reported with ICD-codes, which was the case with our database. We computed each patient's ICISS by multiplying the Survival Risk Ratio (SRR) of each individual injury ICD-code to obtain a final ICISS probability of survival, ranging between 0–1 [9]. Individual SRR have been calculated using large trauma databases and constitute the proportion of survivors in a population with a specific ICD-code. We used ICD-10 SRR computed by Stephensen et al. [10] because at the time of the study this was, as far as we knew, the only database with ICD-10 SRR.

Resource use was defined as any of the following: blood transfusion or major surgery such as brain or spine, thoracic, abdominal, neck, vascular, debridement under general anaesthesia, and critical orthopaedic surgery according to ICD-9-CM-3 procedure codes as reported in the database. A similar definition of resource use has been used in previous studies on injuries and trauma [6], [11]. However, these studies excluded orthopaedic interventions and debridement from their definition of resource use. It was reasonable to include orthopaedic interventions in resource use for this study, because orthopaedic interventions constitute a majority of the interventions performed after an earthquake. Similarly, as extensive debridement is common following earthquakes, in our analyses we included debridement performed under general anaesthesia, which was assumed to indicate extensive debridement.

We categorized patients into three groups according to age: children, adults, and older to allow for stratified analyses. Children were defined as between 0 and 14 years of age, adults were defined as between 15 and 64 years of age, and older were defined as 65 years of age and above. These cut-offs have been used in previous research [4].

### Data management and statistical analysis

Excel software (Microsoft Excel for Mac 2011 v 14.1.4, Microsoft Corporation) was used to clean and prepare the database by correcting erroneous entries and removing variables not needed for subsequent analysis. All statistical analyses were performed using STATA statistical software (STATA 12, StataCorp, Texas). Descriptive statistics were used to characterize sample demographics, and included and excluded patients were compared using cross-tabulation. Kernel-density plots and the Shapiro-Wilk (W) test were used to explore distribution of continuous variables. All continuous variables were found to be non-normally distributed and are thus described using their median, range, and IQR metrics.

We used two methods to assess potential heterogeneity between subgroups. We defined heterogeneity as statistically significant variations in distribution of severe injury, resource use, and serious injury across subgroups. The subgroups considered were male children, female children, male adults, female adults, older males, and older females. Firstly, we calculated proportions, with associated standard errors (SE) and 95% CIs, of patients with severe injury, serious injury, and resource use in each subgroup. We then plotted the proportions with the upper and lower confidence limits and visually assessed for overlapping intervals, both 95% CI and SE. We based this visual assessment on the general rule that when two 95% CIs overlap by one quarter or less of the average CI width then p≈0.05, provided that both samples are larger than ten [12]. Secondly, we assessed for statistically significant heterogeneity using STATA's built in exact tests.

We used nonparametric receiver operating characteristics (ROC) analysis to assess discriminatory accuracy of LOS for identifying severe injury [13]. We decided to use the nonparametric approach over the parametric because our predictor variable was not normally distributed, and hence the parametric approach might have generated biased estimates [14]. The area under the curve (AUC) was used as a measure of discriminatory accuracy. An AUC = 1 was defined as perfect discriminatory accuracy, 0.9<AUC<1 was considered excellent, 0.8<AUC<0.9 good, 0.7<AUC<0.8 adequate, 0.6<AUC<0.7 poor, and AUC<0.6 as no discriminatory accuracy.

The discriminatory accuracy of LOS for predicting serious injury and resource use individually was also analysed. A stratified analysis using age groups and sex as different strata was performed. In addition, sensitivity analyses to test the effect of excluding patients meeting the exclusion criteria were performed. All patients in the database were divided into four groups for sensitivity analyses. Group 1 consisted of included only (not meeting exclusion criteria), group 2 consisted of included and excluded because of coding errors, group 3 consisted of included and excluded because of death or transfer, and group 4 consisted of all patients.

The Youden index (*J*) was used to identify optimal LOS cut off points for significant discriminatory accuracies and was calculated using the formula *J = sensitivity*+*specificity*−*1* [15]. Hence, *J* provides a summary measure of the discriminatory capacity of diagnostic tests at specific cut off points. *J* ranges from −1 to 1. A *J* of −1 indicates a worthless discriminatory capacity, where both sensitivity and specificity are 0%. *J* = 1, on the other hand, indicates a perfect discriminatory capacity where both sensitivity and specificity are 100%. A 5% significance level and a 95% confidence level were used for all statistical tests.

## Results

**Figure 1:**
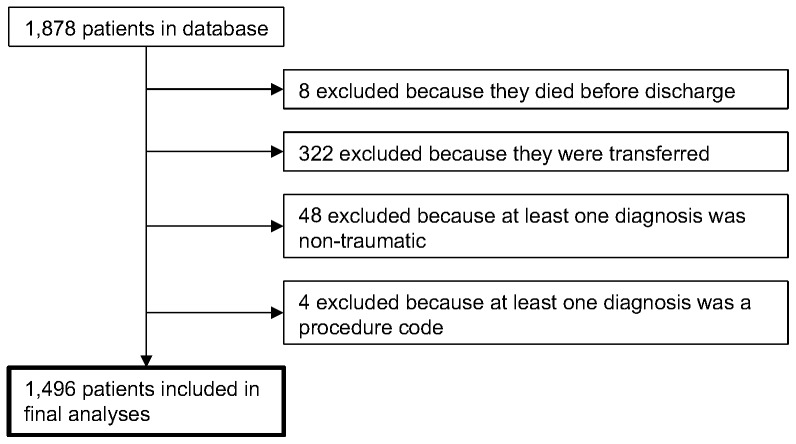
Flow chart describing how the final sample was obtained.

Of the 1,878 patients originally in the database, 1,496 were included in the final analyses (Figure 1). To evaluate potential bias introduced by our exclusion criteria, we compared the demographic characteristics between included and excluded patients in detail (Table 2). Overall, 46.1% (n = 734) of included patients had the primary outcome of severe injury. In terms of secondary outcomes, 26.1% (n = 391) had only resource use and 7% (n = 105) had only serious injury.

**Table 2:** Cross tabulation of demographic characteristics of included and excluded patients.*

		Included patients (n = 1496)	Excluded patients, coding^1^ (n = 52)	Excluded patients, transferred & dead^2^ (n = 330)
Age, years		44 (1–91:32–59.5)	51.5 (6–87:37–71)	40 (1–102:23–60)
Age categories, years (%)	0–14	153 (10.2)	2 (3.9)	50 (15.2)
	15–64	1,067 (71.3)	33 (63.5)	210 (63.6)
	≥65	276 (18.5)	17 (32.7)	70 (21.2)
Female (%)		755 (50.5)	29 (55.8)	157 (47.7)
LOS, days		7 (1–120:4–11)	11.5 (1–72:7–25.5)	4 (1–19:2–7)
Serious injury^3^ (%)		343 (22.9)	9 (17.3)	134 (40.6)
Number of injuries (%)	0	0 (0)	10 (19.2)	2 (0.6)
	1	693 (46.3)	29 (55.8)	99 (30)
	2	690 (46.1)	10 (19.2)	146 (44.2)
	3	97 (6.5)	2 (3.9)	64 (19.4)
	4	13 (0.9)	1 (2)	19 (5.8)
	5	3 (0.2)	0 (0)	0 (0)
Major surgery (%)				
None		871 (58.2)	20 (38.5)	129 (39.1)
Non-orthopaedic		90 (6)	22 (42.3)	51 (15.5)
Critical orthopaedic		534 (35.7)	9 (17.3)	150 (45.5)
Debridement under GA		1 (0.1)	1 (1.9)	0 (0)
Blood transfusion (%)		34 (2.3)	6 (11.5)	42 (12.7)
Resource use^4^ (%)		629 (42.1)	34 (65.4)	205 (62.1)
Severe injury^5^ (%)		734 (49.1)	36 (69.2)	239 (72.4)

* Values are expressed as medians (range:IQR) or proportions (%) where appropriate. Abbreviations: GA  =  General Anaesthesia; ICISS  =  ICD-derived Injury Severity Score; LOS  =  Length of Hospital Stay. ^1^Excluded due to coding errors; ^2^Excluded because patients where either transferred or died before discharge ^3^Defined as ICISS<0.90; ^4^Defined as major surgery (such as brain or spine, thoracic, abdominal, neck, vascular, debridement under GA, or critical orthopaedic surgery) or blood transfusion; ^5^According to either serious injury or resource use definition.

We found no evidence of heterogeneity between subgroups for severe injury (p = 0.2931) or serious injury (p = 0.8929), but we did for resource use (p = 0.0288). Based on these findings, we decided to report ROC results for severe injury and serious injury on overall level, and for resource use on overall and subgroup level. Discriminatory accuracy of LOS for identifying severe injury was poor for severe injury (AUC: 0.622; 95% CI: 0.594–0.650), severe injury excluding critical orthopaedic surgery (COS) (AUC: 0.640; 95% CI: 0.609–0.671), severe injury excluding debridement under general anaesthesia (DGA) (AUC: 0.620; 95% CI: 0.592–0.648), and severe injury excluding COS and DGA (AUC: 0.647 95% CI: 0.616–0.678). It was also poor for serious injury (AUC: 0.635; 95% CI: 0.604–0.666).

Discriminatory accuracy of LOS for identifying resource use ranged from 0.566 to 0.997 (table 3). It was adequate on an overall level when LOS was used to identify patients with resource use excluding COI and DGA (AUC: 0.719 95%; CI: 0.616–0.822). The best discriminatory accuracy was observed in older females, for LOS identifying resource use when COI and DGA were excluded. This discriminatory accuracy was significantly better than good discriminatory accuracy (95% CI: 0.990-1.000). However, it was not significantly different from older males, or older males and females analysed together (p = 0.2482).

**Table 3:** Accuracy measures for stratified ROC analyses, using LOS to identify patients with resource use.*

	Resource use	Excluding COS	Excluding DGA	Excluding COS & DGA 3
All ages	0.612 (0.583–0.640)	0.693 (0.642–0.743)	0.611 (0.583–0.639)	0.719 (0.616–0.822)**
	0.607 (0.567–0.647)	0.678 (0.608–0.748)	0.606 (0.566–0.646)	0.733 (0.578–0.887)**
	0.616 (0.576–0.656)	0.712 (0.640–0.785)**	0.616 (0.576–0.655)	0.709 (0.568–0.850)**
Children (0-14 years)	0.626 (0.531–0.721)	0.655 (0.490–0.821)	0.607 (0.510–0.704)	0.647 (0.391–0.902)
	0.578 (0.423–0.732)	0.570 (0.226–0.914)	0.561 (0.402–0.721)	0.638 (0.148–1.000)
	0.658 (0.535–0.781)	0.714 (0.555–0.873)**	0.634 (0.510–0.759)	0.661 (0.329–0.993)
Adults (15-64 years)	0.605 (0.571–0.638)	0.694 (0.637–0.750)	0.606 (0.573–0.640)	0.671 (0.537–0.805)
	0.620 (0.573–0.667)	0.686 (0.611–0.761)	0.620 (0.573–0.667)	0.686 (0.477–0.895)
	0.589 (0.542–0.637)	0.704 (0.618–0.791)**	0.593 (0.545–0.640)	0.661 (0.482–0.840)
Older (≥65 years)	0.633 (0.568–0.698)	0.733 (0.561–0.905)	0.633 (0.568–0.698)	0.962 (0.905–1.000)**
	0.566 (0.471–0.661)	0.688 (0.470–0.905)	0.566 (0.471–0.661)	0.922 (0.794–1.000)**
	0.694 (0.605–0.784)	0.831 (0.520–1.000)**	0.694 (0.605–0.784)	0.997 (0.990–1.000)**

* Data is reported as AUC (95% CI); **Adequate or higher discriminatory accuracy. Abbreviations: AUC  =  Area under the curve; COS  =  Critical Orthopaedic Surgery; DGA  =  Debridement under General Anaesthesia; ICISS  =  ICD-derived Injury Severity Score; ROC  =  Receiver operating characteristics. ^1^According to either serious injury or resource use definition; ^2^Defined as major surgery (such as brain or spine, thoracic, abdominal, neck, vascular, debridement under general anaesthesia, or critical orthopaedic surgery) or blood transfusion; ^3^Defined as ICISS<0.90.

The optimal LOS cut off points for identifying patients with resource use excluding COI and DGA on an overall level was ≥11 days (maximum *J* = 0.38), with a corresponding sensitivity and specificity of 64.7% and 73.1% respectively. For older people of both sexes the optimal cut off was ≥12 days (maximum *J* = 0.81), with a corresponding sensitivity and specificity of 83.3% and 81.1% respectively. For older males the optimal cut off was ≥13 days (maximum *J* = 0.79), with a corresponding sensitivity and specificity of 100% and 79.1% respectively. For older females the optimal cut off was ≥67 days (maximum *J* = 0.99), with a corresponding sensitivity and specificity of 100% and 99.2% respectively.

Sensitivity analyses comparing group 1 with groups 2-4 revealed worse discriminatory accuracy of LOS for all outcomes in groups 3 and 4. For group 2, ROC AUC did not change significantly. The ROC AUC range was 0.506–0.957 in group 2, 0.393–0.709 in group 3, and 0.499–0.792 in group 4 (Table S1, Figure S1). It is likely that the different discriminatory accuracy for groups 3 and 4 is due to the arbitrary LOS of dead and transferred patients.

## Discussion

Our findings show that overall LOS is a poor indicator for injury severity in survivors of the 2008 Wenchuan earthquake. Instead, LOS showed adequate discriminatory accuracy when used to distinguish patients who received major non-orthopaedic surgery or blood transfusion and those who did not. In addition, the discriminatory accuracy for older survivors was almost perfect when LOS was used to distinguish patients who received major non-orthopaedic surgery or blood transfusion and those who did not. We also found differences between included and excluded patients, most likely reflecting the fact that more severely injured patients were transferred to more advanced centres.

Intuitively, it seems reasonable to assume that LOS reflects injury severity. Patients with more severe injuries are expected to stay in the hospital longer than patients with less severe injuries. Newgard et al. validated LOS as a proxy indicator for injury severity in trauma survivors [4]. Our findings do not corroborate with those of the Newgard et al. study. However, other reports support our results [16], [17].

Unlike Newgard et al., we sampled an older population with a longer LOS. In addition, our sample population had higher proportions of people with serious injury, receiving blood transfusion, fulfilling resource use definition, and being classified as having severe injury. It is possible that these differences in our study and that of Newgard et al. account for some of the discrepancies in our results, as injury severity is not the only factor that affects LOS. For example, patient age is significantly associated with LOS [18]. Type of injury could influence the willingness of doctors to discharge patients. For instance, complex fractures that would not require hospitalization normally may make it difficult for a patient to recover in a house that has been affected by an earthquake. Additionally, patients in our sample may have remained hospitalized longer because they had to travel long distances to return home, had no home to return to, or for other reasons beyond the aim of this study.

When LOS was assessed as an indicator for resource in the absence of critical orthopaedic interventions, we found that the discriminatory accuracy increased. This increase may be because patients with orthopaedic conditions are not as severely injured as patients requiring other major surgery. The increase in discriminatory accuracy suggests an association between LOS, major non-orthopaedic surgery, and blood transfusion that warrants further investigation. This is particularly interesting because receiving a blood transfusion has been associated with longer LOS in trauma patients, regardless of injury severity and other potential confounders [19].

The poor correlation between LOS and severe injury in our study may be attributed to the chaos caused by the earthquake initially. People's Hospital of Deyang City was flooded with patients and health care staff may have been forced to prioritize differently than under normal circumstances. Our finding that the discriminatory accuracy was higher for resource use when critical orthopaedic interventions and debridement were excluded supports this hypothesis. For instance, patients who received major interventions might have had a longer LOS than they would have under normal circumstances, whereas patients who may have remained hospitalized longer in the non-disaster setting could have been discharged earlier.

Foreign medical teams often arrive in low- and middle-income settings to provide additional aid after earthquakes. Although this practise saves lives that may not otherwise be spared, it has been repeatedly criticized as such teams often arrive too late to provide life-saving trauma care [20], [21], are uncoordinated [22], lack professional standards, lack appropriately skilled staff, use inappropriate interventions [23], and have poor outcomes [24]. Additionally, the accountability of foreign medical teams has been questioned [25].

The need for increased transparency and accountability of international medical relief after disasters is being addressed through evidence-based research. Developing uniform methods for medical response to disasters could increase the efficiency of foreign responders as well as that of native medical teams. Thus, The Lancet published a series on evidence for complex emergencies in 2004 [26]. Following the 2004 tsunami in Indonesia, the Cochrane Collaboration initiated the Evidence Aid project, which aims to use systematic reviews to generate evidence to improve the response to disasters [27]. Furthermore, the World Health Organization has recently established a working group to create a register and accreditation system for foreign medical teams [28].

Our findings provide a tool for retrospective research on major non-orthopaedic surgery and blood transfusions following earthquakes that does not require collection of detailed data on the intervention level. Data on LOS can be collected through interviews with health care staff and patients, and can then be used for planning purposes, including resource allocation. However, more research in other disaster settings is needed before LOS can be used as a proxy indicator for major non-orthopaedic surgery and blood transfusions. For instance, it should be determined if LOS can be used to compare resource use in different hospitals or to monitor resource use over time. Cryer et al. showed that LOS is not a stable proxy indicator in non-disaster areas of high income [29]. Whether this finding holds true in low- and middle-income disaster settings is not know.

### Limitations

This study has several limitations. Most importantly, the database was collated from patient files recorded in the immediate post-earthquake setting. This is problematic, as injury diagnoses are not always correctly recorded, even in normal health care setting. Therefore, it can be reasonably assumed that some diagnoses in our dataset were incorrectly recorded. However, due to the difficultly of assessing such errors, it is impossible to adjust for this.

Another limitation is that we are not sure about the generalizability of our findings. People's Hospital of Deyang City was, considering the circumstances, well functioning. If our findings hold true for hospitals that are more severely affected requires further research. Despite its limitations, the database from the 2008 Wenchuan earthquake is one of the most comprehensive collations of data from an injured population following an earthquake. Use of this database offers a unique opportunity to produce new knowledge in disaster medicine.

## Conclusions

Our study shows that as an overall measure, LOS is not an adequate proxy indicator for injury severity in earthquake survivors. However, our LOS appears to be an adequate proxy indicator for major non-orthopaedic surgery and blood transfusion after an earthquake. This finding holds particular promise as a potential tool for retrospective research on surgical resource use after earthquakes, if detailed hospital records are not available.

## Supporting Information

Figure S1 **Graphical comparison of all ROC analyses.**
(PDF)

Table S1 **Complete reference over all ROC analyses.**
(XLSX)

## References

[1] Centre for Research on the Epidemiology of Disasters (2012) EM-DAT: The International Disaster Database. Brussels: Centre for Research on the Epidemiology of Disasters (CRED).

[2] Alexander D (1996) The health effects of earthquakes in the mid-1990s. Disasters 20: 231–247. 8854459 10.1111/j.1467-7717.1996.tb01036.x

[3] Bartels SA, Vanrooyen MJ (2011) Medical complications associated with earthquakes. Lancet 379: 748–757. 22056246 10.1016/S0140-6736(11)60887-8

[4] Newgard CD, Fleischman R, Choo E, Ma OJ, Hedges JR, et al (2010) Validation of length of hospital stay as a surrogate measure for injury severity and resource use among injury survivors. Acad Emerg Med 17: 142–150. 20370743 10.1111/j.1553-2712.2009.00647.x PMC4715859

[5] World Health Organization (2008) Communicable disease risk assessment and interventions: Sichuan earthquake: the People's Republic of China. Geneva, Switzerland: World Health Organization.

[6] Newgard CD, Hedges JR, Diggs B, Mullins RJ (2008) Establishing the need for trauma center care: anatomic injury or resource use? Prehosp Emerg Care 12: 451–458. 18924008 10.1080/10903120802290737

[7] Diggs BS, Mullins RJ, Hedges JR, Arthur M, Newgard CD (2008) Proportion of seriously injured patients admitted to hospitals in the US with a high annual injured patient volume: a metric of regionalized trauma care. J Am Coll Surg 206: 212–219. 18222372 10.1016/j.jamcollsurg.2007.08.019

[8] Rutledge R, Osler T, Emery S, Kromhout-Schiro S (1998) The end of the Injury Severity Score (ISS) and the Trauma and Injury Severity Score (TRISS): ICISS, an International Classification of Diseases, ninth revision-based prediction tool, outperforms both ISS and TRISS as predictors of trauma patient survival, hospital charges, and hospital length of stay. J Trauma 44: 41–49. 9464748 10.1097/00005373-199801000-00003

[9] Osler T, Rutledge R, Deis J, Bedrick E (1996) ICISS: an international classification of disease-9 based injury severity score. J Trauma 41: 380–386; discussion 386–388. 8810953 10.1097/00005373-199609000-00002

[10] Stephenson S, Langley J, Henley G, Harrison J (2003) Diagnosis-based injury severity scaling-a method using Australian and New Zealand hospital data coded to ICD-10-AM. Canberra: Australian Institute of Health and Welfare. 10.1136/ip.2004.005561 PMC1730146 15583261

[11] Baxt WG, Jones G, Fortlage D (1990) The trauma triage rule: a new, resource-based approach to the prehospital identification of major trauma victims. Ann Emerg Med 19: 1401–1406. 2240753 10.1016/s0196-0644(05)82608-3

[12] Cumming G, Finch S (2005) Inference by eye-Confidence intervals and how to read pictures of data. American Psychologist 60: 170–180. 15740449 10.1037/0003-066X.60.2.170

[13] Lett RR, Hanley JA, Smith JS (1995) The comparison of injury severity instrument performance using likelihood ratio and ROC curve analyses. J Trauma 38: 142–148. 7745645 10.1097/00005373-199501000-00032

[14] Colak E, Mutlu F, Bal C, Oner S, Ozdamar K, et al (2012) Comparison of semiparametric, parametric, and nonparametric ROC analysis for continuous diagnostic tests using a simulation study and acute coronary syndrome data. Comput Math Methods Med 2012: 698320. 22844346 10.1155/2012/698320 PMC3395260

[15] Youden WJ (1950) Index for Rating Diagnostic Tests. Cancer 3: 32–35. 15405679 10.1002/1097-0142(1950)3:1<32::aid-cncr2820030106>3.0.co;2-3

[16] Schluter PJ, Cameron CM, Davey TM, Civil I, Orchard J, et al (2009) Using Trauma Injury Severity Score (TRISS) variables to predict length of hospital stay following trauma in New Zealand. N Z Med J 122: 65–78. 19834524

[17] Fern KT, Smith JT, Zee B, Lee A, Borschneck D, et al (1998) Trauma patients with multiple extremity injuries: resource utilization and long-term outcome in relation to injury severity scores. J Trauma 45: 489–494. 9751538 10.1097/00005373-199809000-00010

[18] Weingarten MS, Wainwright ST, Sacchetti AD (1988) Trauma and Aging Effects on Hospital Costs and Length of Stay. Ann Emerg Med 17: 10–14. 3337401 10.1016/s0196-0644(88)80494-3

[19] Malone DL, Dunne J, Tracy JK, Putnam AT, Scalea TM, et al (2003) Blood transfusion, independent of shock severity, is associated with worse outcome in trauma. Journal of Trauma-Injury Infection and Critical Care 54: 898–905. 10.1097/01.TA.0000060261.10597.5C 12777902

[20] von Schreeb J, Riddez L, Samnegard H, Rosling H (2008) Foreign field hospitals in the recent sudden-onset disasters in Iran, Haiti, Indonesia, and Pakistan. Prehosp Disaster Med 23: 144–151; discussion 152-143. 18557294 10.1017/s1049023x00005768

[21] de Ville de Goyet C (2007) Health lessons learned from the recent earthquakes and Tsunami in Asia. Prehosp Disaster Med 22: 15–21. 17484358 10.1017/s1049023x00004283

[22] Abolghasemi H, Radfar MH, Khatami M, Nia MS, Amid A, et al (2006) International medical response to a natural disaster: lessons learned from the Bam earthquake experience. Prehosp Disaster Med 21: 141–147. 16892878 10.1017/s1049023x00003599

[23] Jobe K (2011) Disaster relief in post-earthquake Haiti: unintended consequences of humanitarian volunteerism. Travel Med Infect Dis 9: 1–5. 21130039 10.1016/j.tmaid.2010.10.006

[24] Roy N, Shah H, Patel V, Bagalkote H (2005) Surgical and psychosocial outcomes in the rural injured-a follow-up study of the 2001 earthquake victims. Injury-International Journal of the Care of the Injured 36: 927–934. 10.1016/j.injury.2005.02.017 15979621

[25] Birnbaum ML (2011) Account-ability. Prehosp Disaster Med 26: 77–78. 21888726 10.1017/S1049023X11000173

[26] Sharp D (2004) Hearts and minds, from Darfur to locusts. Lancet 364: 1741–1742. 15541436 10.1016/S0140-6736(04)17417-5

[27] Clarke M (2008) Evidence Aid--from the Asian tsunami to the Wenchuan earthquake. J Evid Based Med 1: 9–11. 21348967 10.1111/j.1756-5391.2008.00007.x

[28] Redmond AD, O'Dempsey TJ, Taithe B (2011) Disasters and a register for foreign medical teams. Lancet 377: 1054–1055. 21440794 10.1016/S0140-6736(11)60319-X

[29] Cryer C, Gulliver P, Langley JD, Davie G (2010) Is length of stay in hospital a stable proxy for injury severity? Injury Prevention 16: 254–260. 20587811 10.1136/ip.2009.023903

