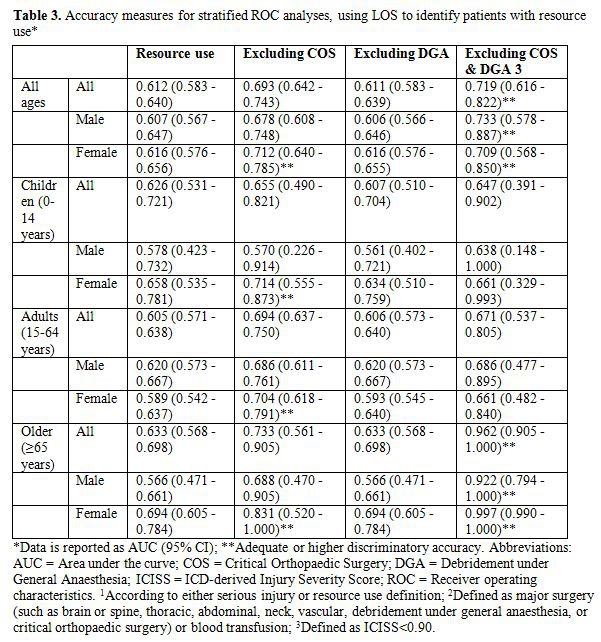# Correction: Hospital Stay as a Proxy Indicator for Severe Injury in Earthquakes: A Retrospective Analysis

**DOI:** 10.1371/annotation/4c2d73d0-d2e8-4efc-947c-adbf4059b81e

**Published:** 2013-10-24

**Authors:** Lu-Ping Zhao, Martin Gerdin, Lina Westman, Jose Manuel Rodriguez-Llanes, Qi Wu, Barbara van den Oever, Liang Pan, Manuel Albela, Gao Chen, De-Sheng Zhang, Debarati Guha-Sapir, Johan von Schreeb

In Table 3, the column detailing gender was missing. Please view the corrected Table 3 here: 

**Figure:**