# Supplementary material for: Hospital Stay as a Proxy Indicator for Severe Injury in Earthquakes: A Retrospective Analysis
Source: PLoS One. 2013 Apr 9;8(4):e61371. doi: 10.1371/journal.pone.0061371 (PMC3621831; doi:10.1371/journal.pone.0061371)
Supplement: Figure S1 — Graphical comparison of all ROC analyses. (PDF) [file pone.0061371.s001.pdf]

All ages, both sexes

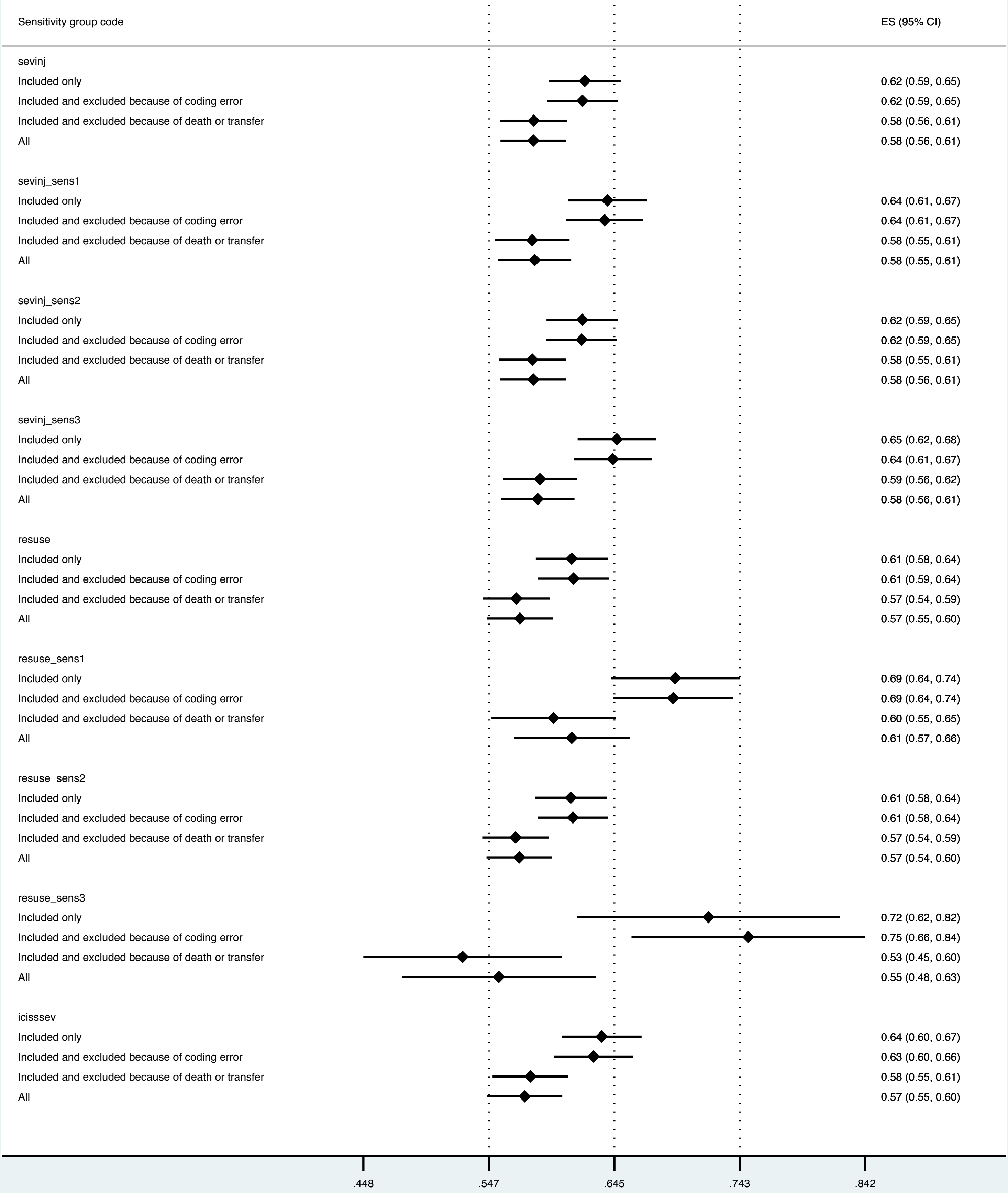

sevinj = Severe injury, according to either serious injury or resource use definition;
sevinj_sens1 = Severe injury, excluding critical orthopaedic interventions from resource use definition;
sevinj_sens2 = Severe injury, excluding debridements under general anaesthesia from resource use definition;
sevinj_sens3 = Severe injury, excluding critical orthopaedic interventions and debridements under general anaesthesia from resource use definition;
resuse = Resource use; resuse_sens1 = Resource use, excluding critical orthopaedic interventions;
resuse_sens2 = Resource use, excluding debridements under general anaesthesia;
resuse_sens3 = excluding critical orthopaedic interventions and debridements under general anaesthesia;
icisssev = Serious injury.
Resource use was defined as major surgery (such as brain or spine, thoracic, abdominal, neck, vascular, debridement under general anaesthesia, or critical orthopaedic surgery) or blood transfusion.
Serious injury was defined as ICISS<0.90

All ages, males

Sensitivity group code	ES (95% CI)
sevinj	
Included only	0.62 (0.58, 0.66)
Included and excluded because of coding error	0.62 (0.58, 0.66)
Included and excluded because of death or transfer	0.58 (0.54, 0.62)
All	0.58 (0.54, 0.62)
sevinj_sens1	
Included only	0.65 (0.61, 0.69)
Included and excluded because of coding error	0.65 (0.60, 0.69)
Included and excluded because of death or transfer	0.58 (0.54, 0.62)
All	0.58 (0.54, 0.62)
sevinj_sens2	
Included only	0.62 (0.58, 0.66)
Included and excluded because of coding error	0.62 (0.58, 0.66)
Included and excluded because of death or transfer	0.58 (0.54, 0.62)
All	0.58 (0.54, 0.62)
sevinj_sens3	
Included only	0.66 (0.62, 0.70)
Included and excluded because of coding error	0.65 (0.61, 0.69)
Included and excluded because of death or transfer	0.58 (0.54, 0.63)
All	0.58 (0.54, 0.62)
resuse	
Included only	0.61 (0.57, 0.65)
Included and excluded because of coding error	0.61 (0.57, 0.65)
Included and excluded because of death or transfer	0.56 (0.52, 0.60)
All	0.56 (0.53, 0.60)
resuse_sens1	
Included only	0.68 (0.61, 0.75)
Included and excluded because of coding error	0.68 (0.61, 0.74)
Included and excluded because of death or transfer	0.59 (0.52, 0.65)
All	0.60 (0.54, 0.66)
resuse_sens2	
Included only	0.61 (0.57, 0.65)
Included and excluded because of coding error	0.61 (0.57, 0.65)
Included and excluded because of death or transfer	0.56 (0.52, 0.60)
All	0.56 (0.53, 0.60)
resuse_sens3	
Included only	0.73 (0.58, 0.89)
Included and excluded because of coding error	0.76 (0.62, 0.90)
Included and excluded because of death or transfer	0.55 (0.43, 0.66)
All	0.57 (0.46, 0.68)
icisssev	
Included only	0.65 (0.61, 0.69)
Included and excluded because of coding error	0.64 (0.60, 0.68)
Included and excluded because of death or transfer	0.58 (0.54, 0.62)
All	0.57 (0.53, 0.61)

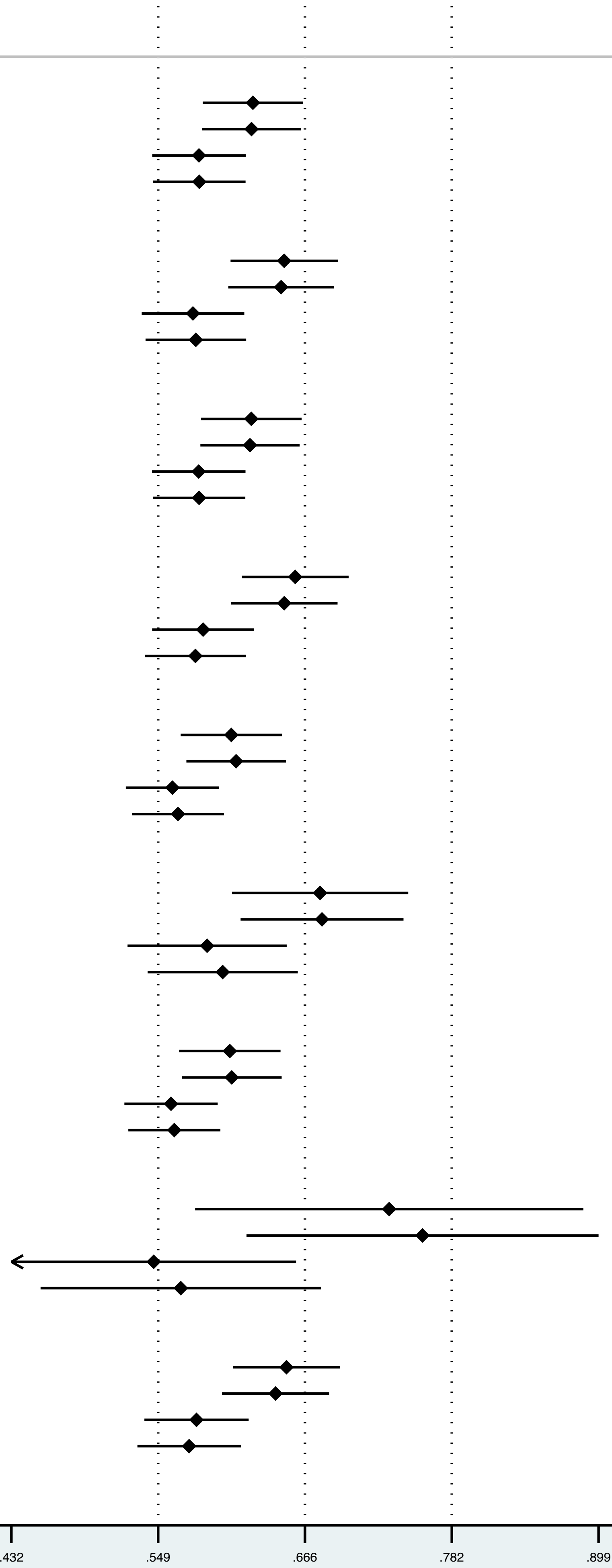

Please see page 1 for definitions

All ages, females

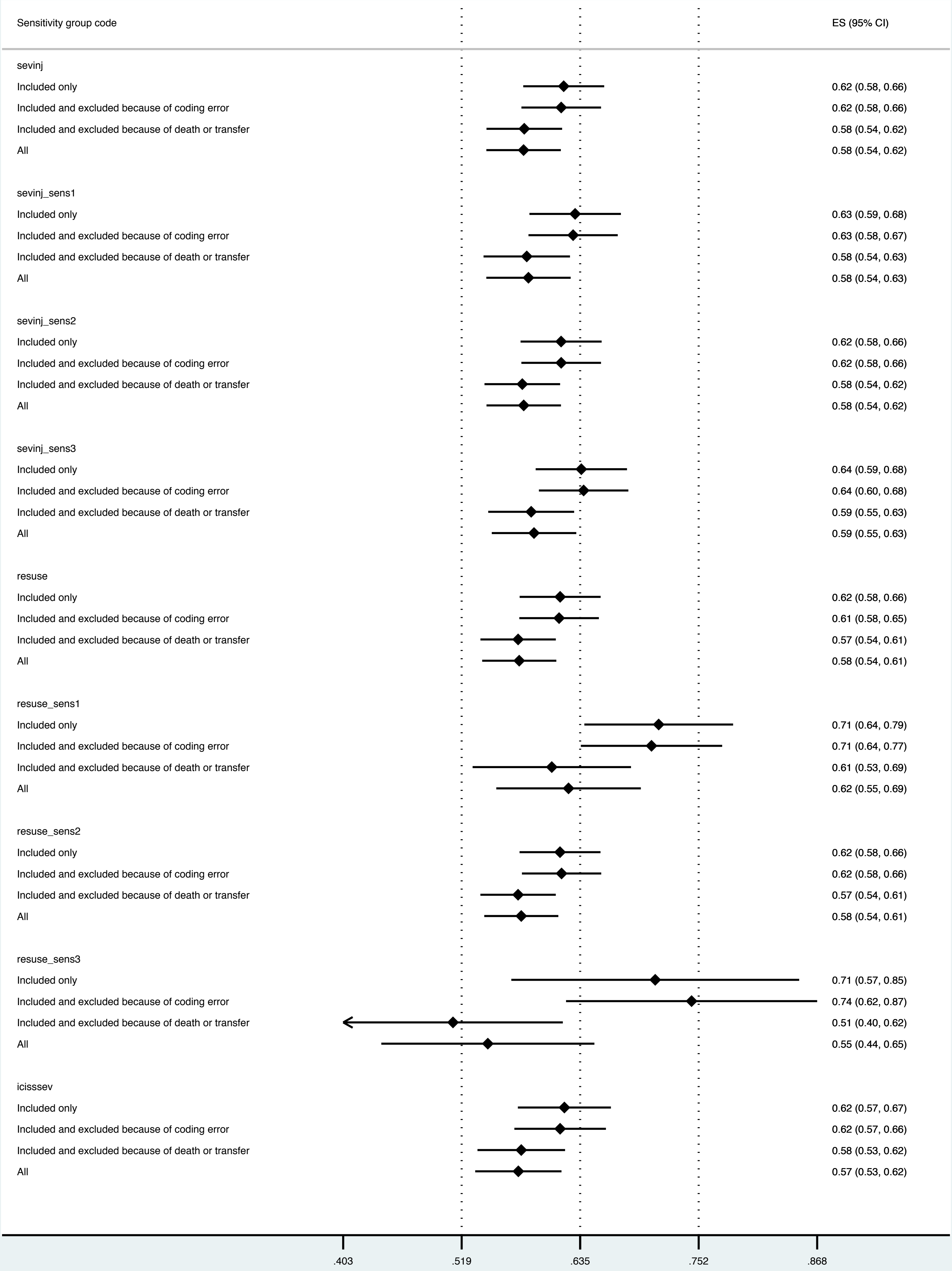

Please see page 1 for definitions

0-14, both sexes

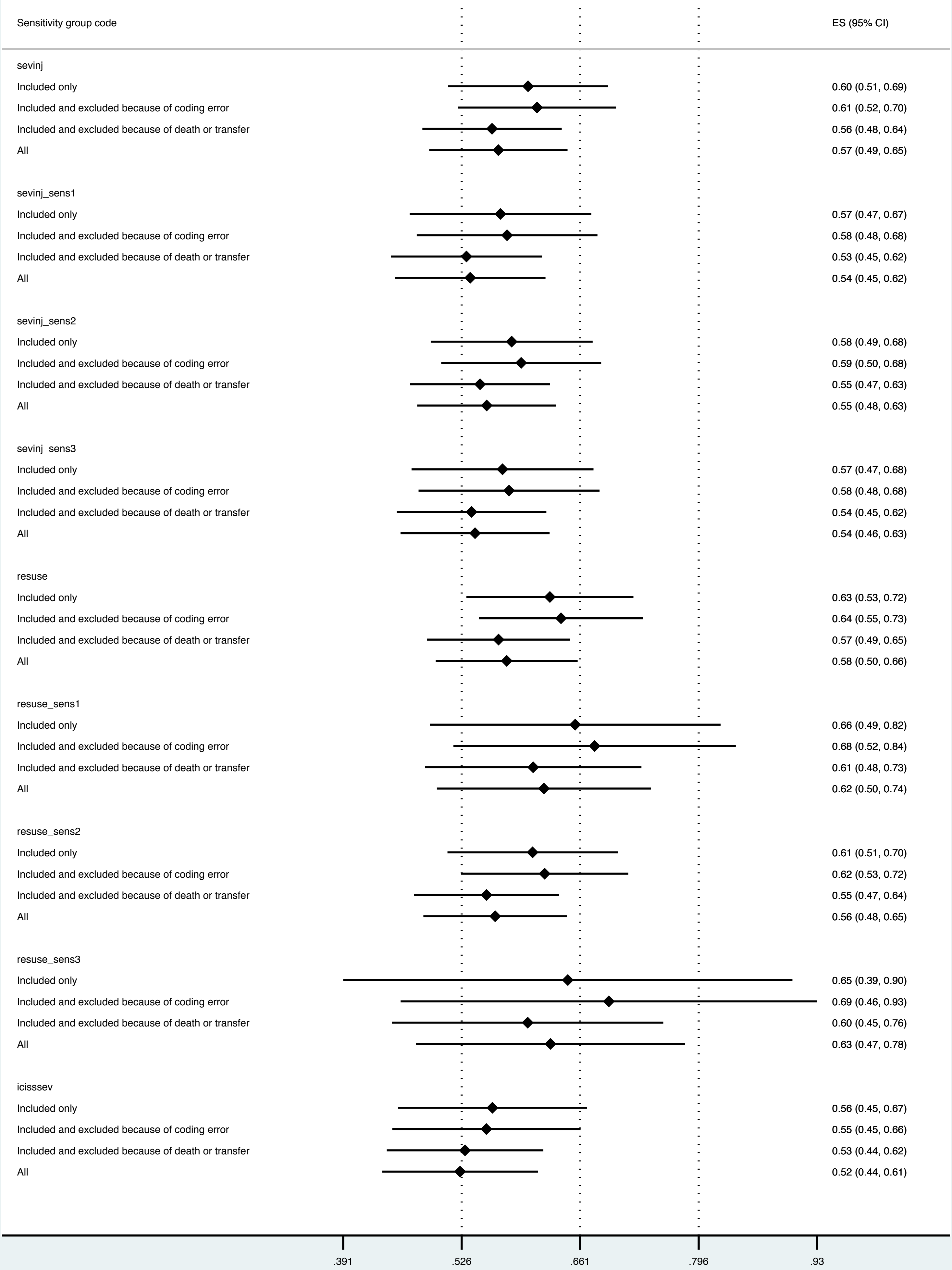

Please see page 1 for definitions

0-14, males

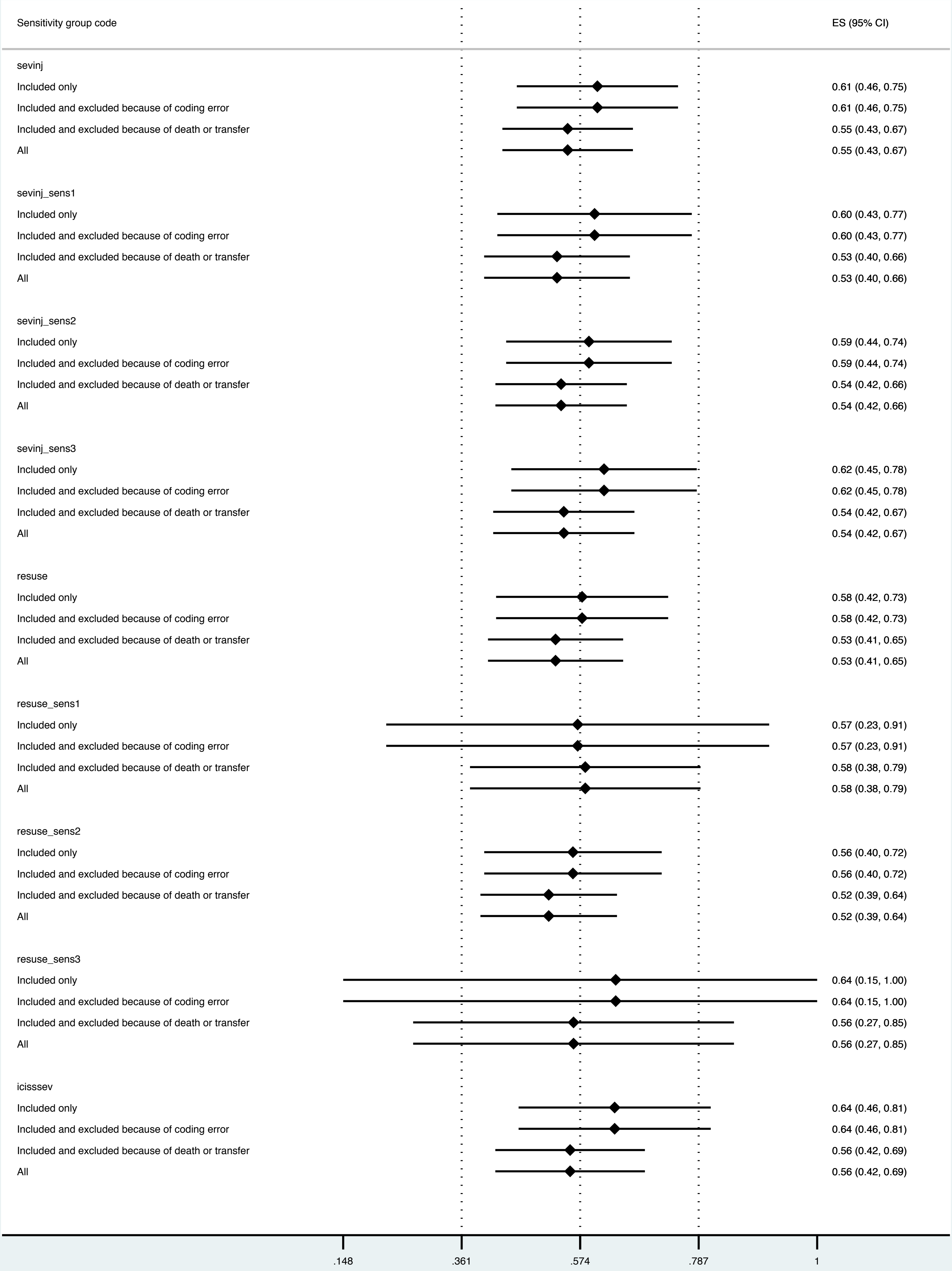

Please see page 1 for definitions

0-14, females

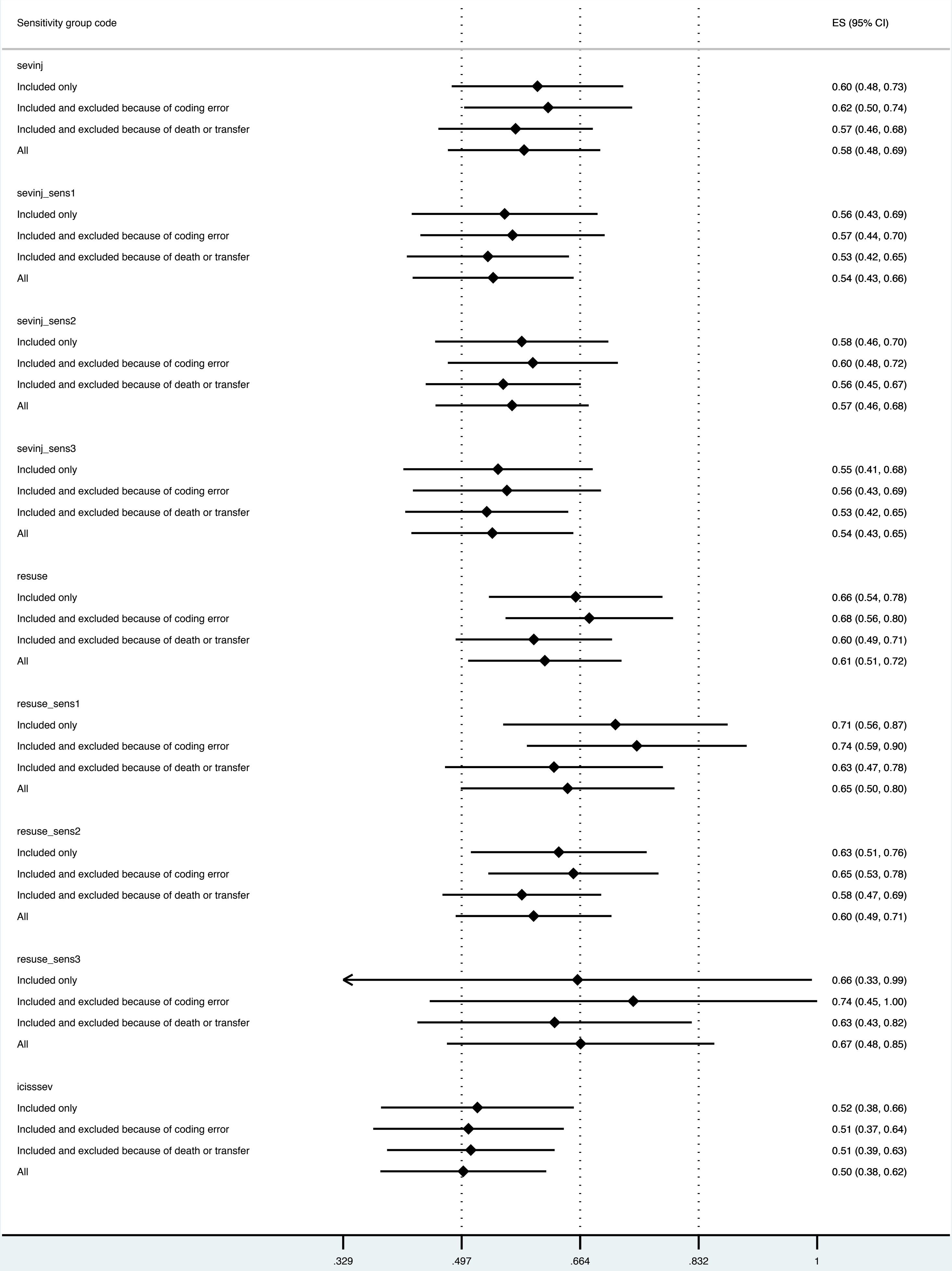

Please see page 1 for definitions

15-64, both sexes

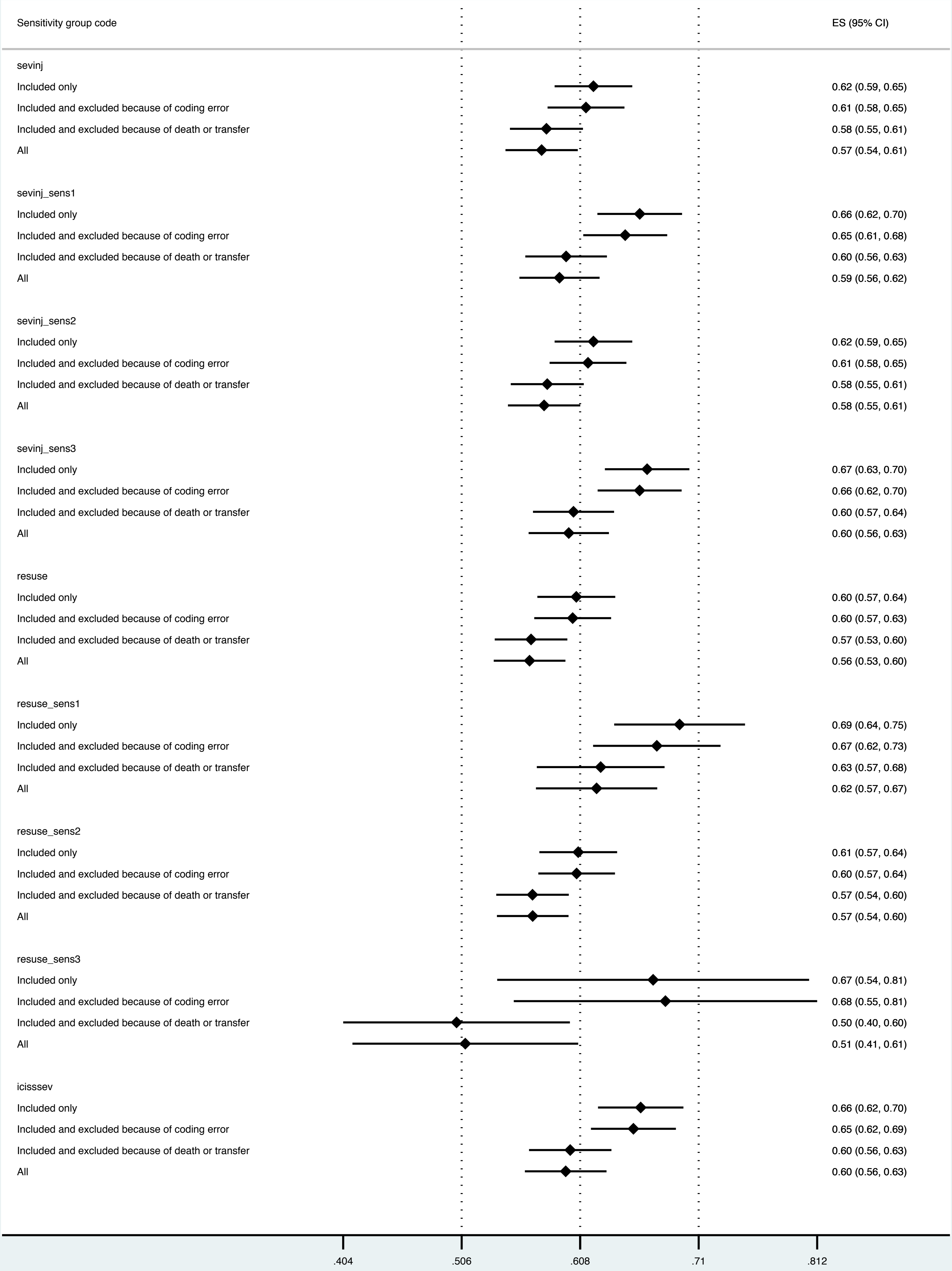

Please see page 1 for definitions

15-64, males

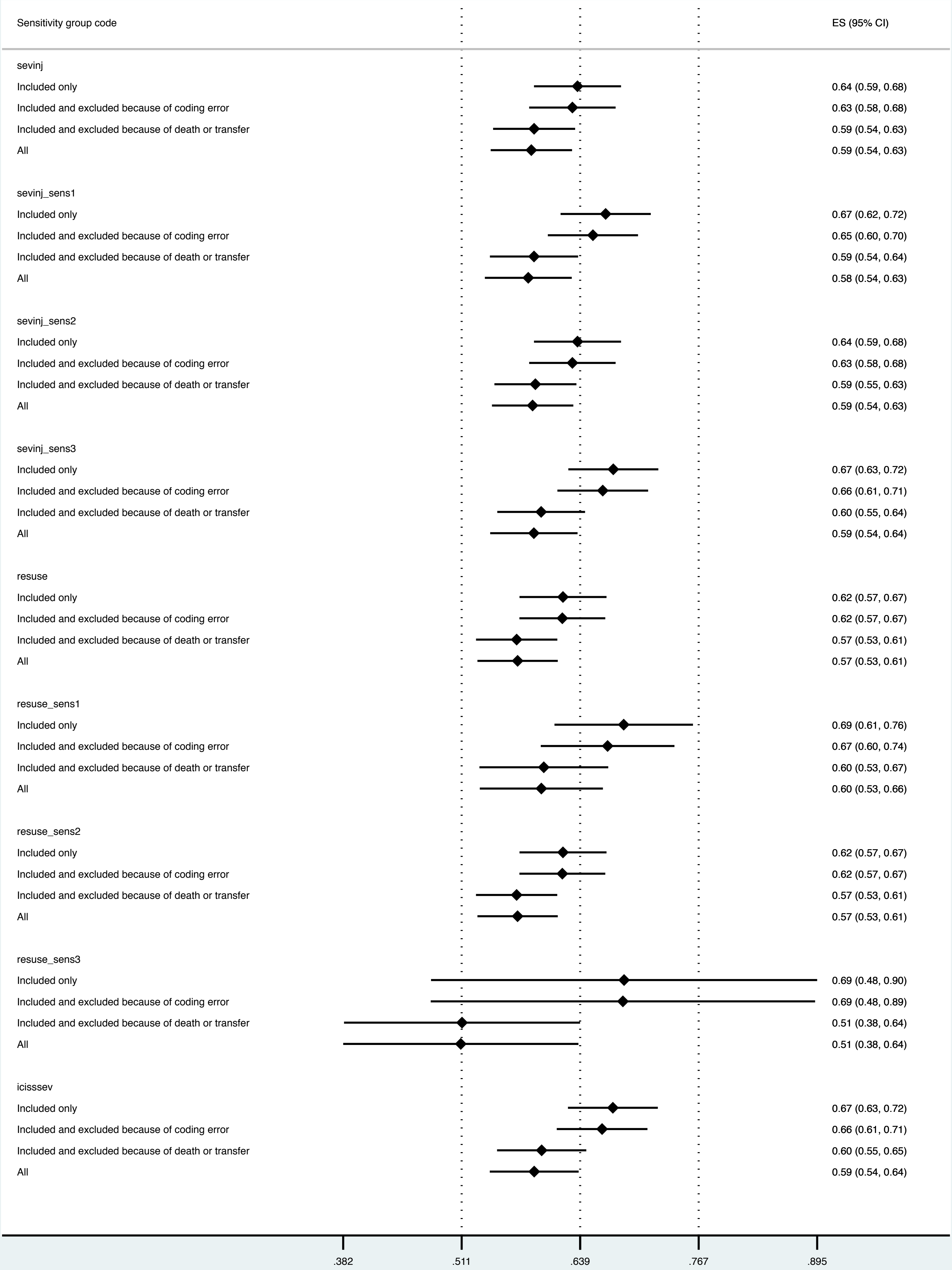

Please see page 1 for definitions

15-64, females

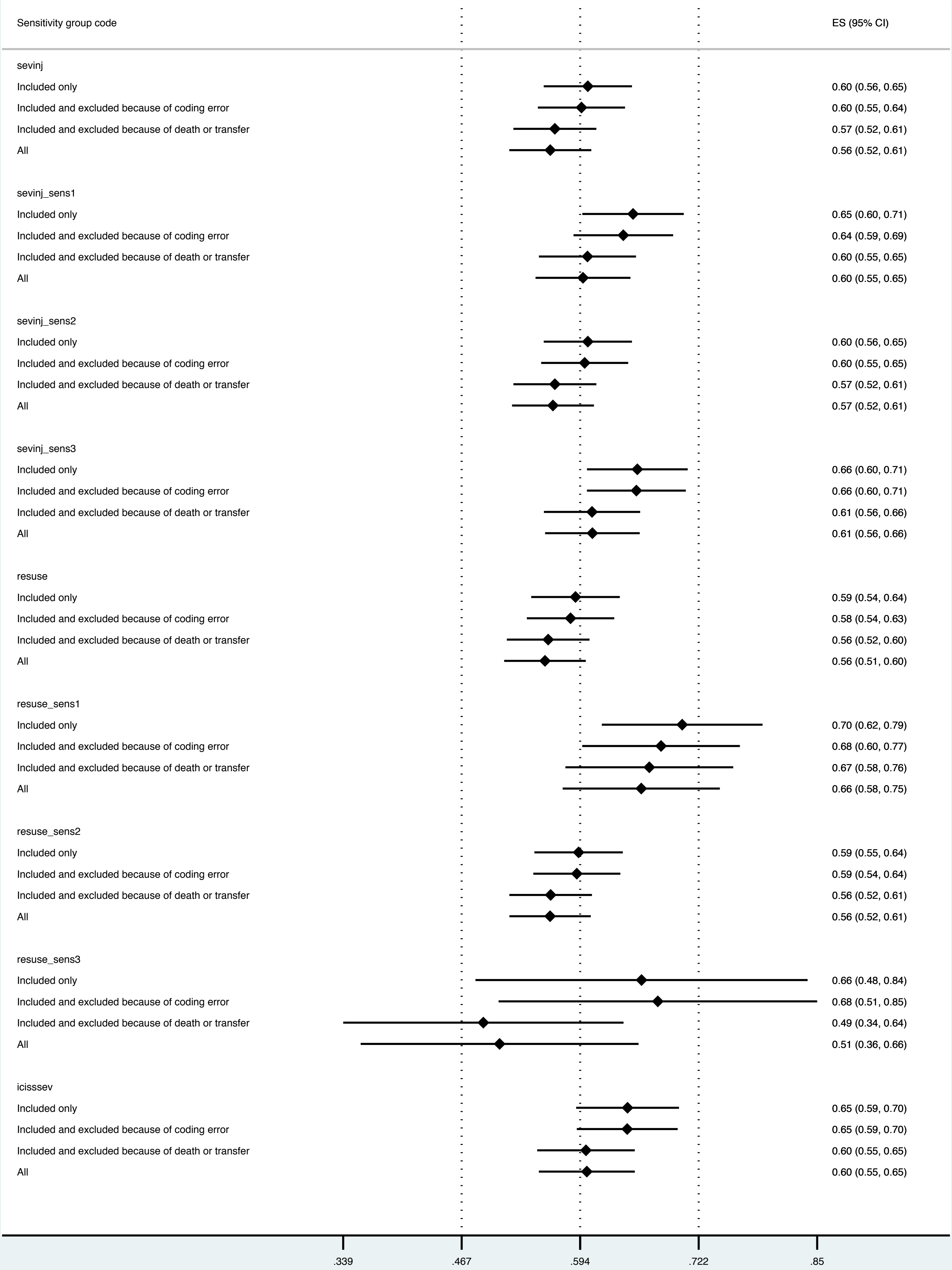

Please see page 1 for definitions

>=65, both sexes

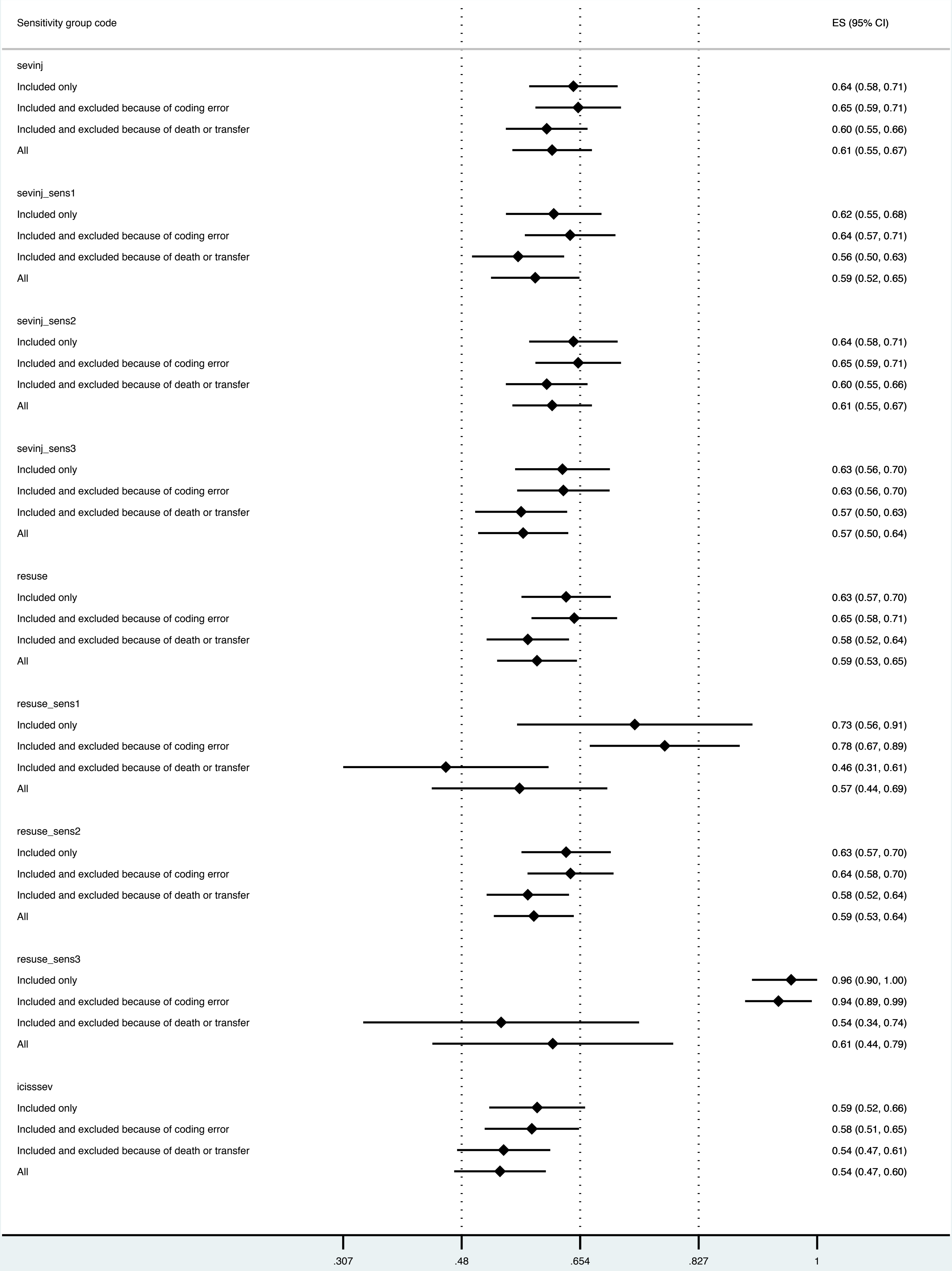

Please see page 1 for definitions

>=65, males

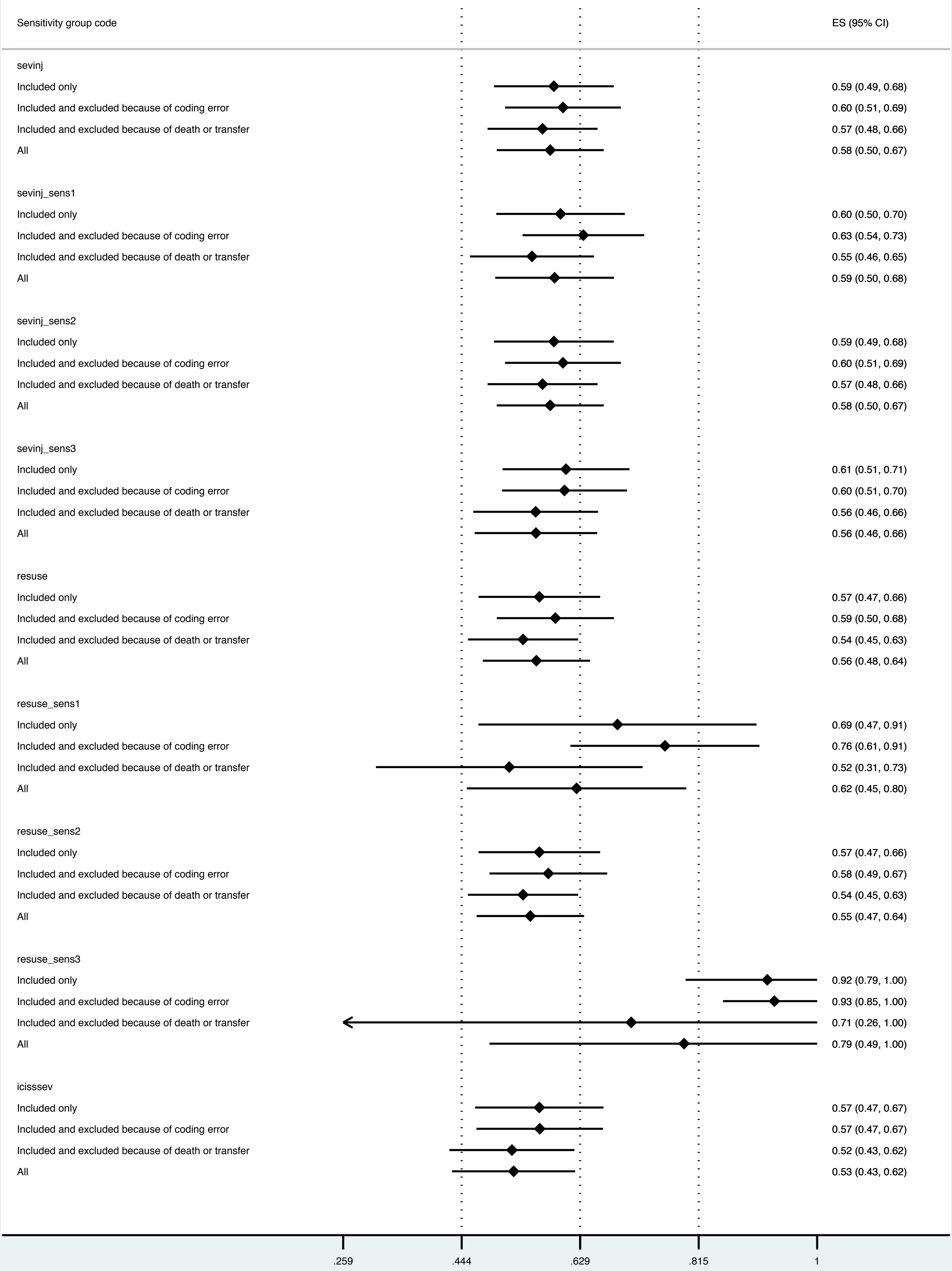

Please see page 1 for definitions

>=65, females

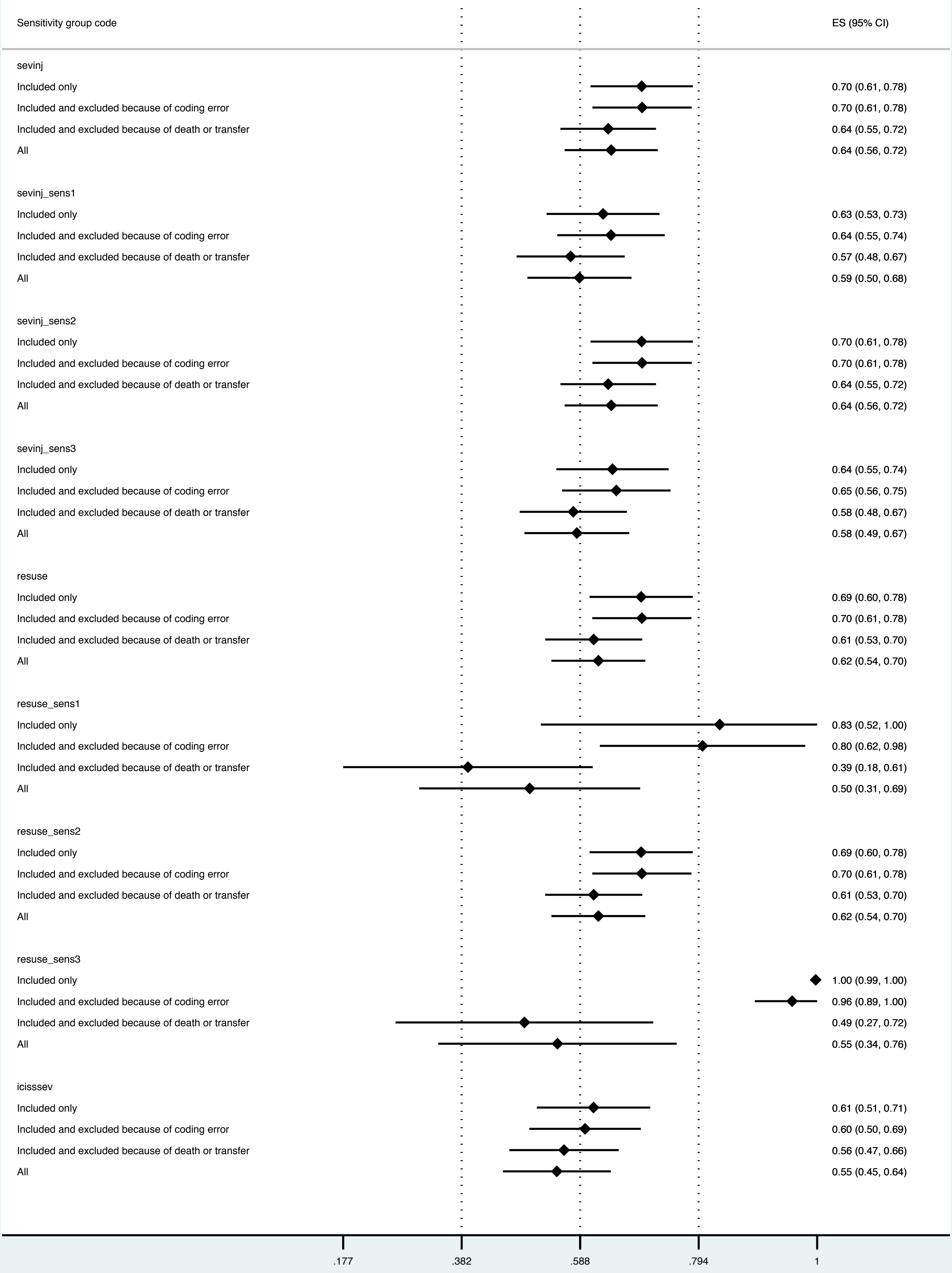

Please see page 1 for definitions
